# Advancements in bladder cancer treatment: The synergy of radiation and immunotherapy

**DOI:** 10.18632/oncotarget.28723

**Published:** 2025-05-19

**Authors:** Nazmul Hasan, Daniel Yang, Spencer Gibson, Barbod Khaleghi, Rozhan Ziari, Arash Rezazadeh Kalebasty

**Affiliations:** ^1^Department of Medicine, University of California, Irvine, CA 92868, USA; ^2^Chao Family Comprehensive Cancer Center, Division of Hematology and Oncology, University of California, Irvine, CA 92868, USA

**Keywords:** bladder cancer, immunotherapy, radiation, microenvironment, abscopal

## Abstract

Different treatment strategies are required for the non-muscle-invasive, muscle-invasive, and metastatic stages of bladder cancer. Standard treatments include surgery, chemotherapy, and radiation; however, they have their limitations. New discoveries have shown that combining immunotherapy and radiation treatment may improve patient outcomes. Radiation therapy promotes immunogenic cell death, which leads to antigen release and immune cell activation, whereas immunotherapy enhances the immune system’s ability to recognize and destroy cancer cells by targeting checkpoint pathways like PD-1/PD-L1 and CTLA-4. This review examines the synergistic mechanisms of diverse modalities, focusing on their capacity to alter the tumor microenvironment and elicit systemic anti-tumor responses, such as the abscopal effect. Key clinical trials, such as BTCRC-GU15-023 and ANZUP, have demonstrated the efficacy and safety of combining these medications. However, difficulties persist, such as overlapping toxicities, unpredictability in patient responses, and a lack of accurate patient selection markers. Large-scale randomized trials are needed in the future to fine-tune treatment procedures, minimize toxicity, and validate predictive biomarkers such as PD-L1 expression and tumor mutation burden. By addressing these hurdles, the combination of radiation treatment and immunotherapy has the potential to change the bladder cancer therapeutic landscape.

## INTRODUCTION

With an expected 85,000 new cases and 19,000 linked deaths in 2020, bladder cancer and ureteral cancer rank as major health issues for the United States [1]. The disease affects men at a higher rate than women [1]. Occupational exposure to aromatic amines [2], chronic urinary tract infections [3, 4], tobacco use [5–7], pelvic radiation [7, 8], and hereditary non-polyposis colorectal cancer (HNPCC) syndrome [9] are among the numerous risk factors that contribute to the development of bladder cancer. 90% of bladder malignancies are urothelial carcinomas [10]. Bladder cancer is broadly classified into three categories: non-muscle-invasive, muscle-invasive, and metastatic. Each category necessitates unique treatment strategies [10] (Table 1). Notwithstanding these established techniques, a substantial unmet requirement persists in enhancing treatment for advanced and recurring bladder cancer. Conventional medicines frequently fail to provide lasting responses, underscoring the need for innovative combination tactics that can combat tumor resistance and improve long-term results.

**Table 1:** Treatment of non-muscle-invasive and muscle-invasive bladder cancer

	Non-muscle-invasive bladder cancer (NMIBC)	Muscle-invasive bladder cancer (MIBC)	References
**Primary Treatment**	TURBT (Transurethral Resection of Bladder Tumor)	Radical cystectomy with neoadjuvant cisplatin-based chemotherapy or bladder-preserving trimodal therapy (TMT)	[11, 13, 14]
**Adjuvant Treatment**	Intravesical BCG, Pembrolizumab for BCG-unresponsive patients	Adjuvant nivolumab for high-risk cases	[11, 12, 15]
**Metastatic Treatment**	Not applicable	Pembrolizumab + Enfortumab Vedotin	[15, 16]

Abbreviations: NMIBC: Non-Muscle-Invasive Bladder Cancer; MIBC: Muscle-Invasive Bladder Cancer; TURBT: Transurethral Resection of Bladder Tumor; TMT trimodal therapy; BCG: Bacillus Calmette-Guerin.

### Standard of care

Non-muscle-invasive bladder cancer (NMIBC) accounts for about 70% of instances, with muscle-invasive bladder cancer (MIBC) accounting for the remaining 30%. Each type of bladder cancer necessitates a distinct treatment approach [10]. The treatment for NMIBC typically entails transurethral resection of the bladder tumor (TURBT), followed by intravesical therapy and surveillance [11]. Intravesical bacillus Calmette-Guerin (BCG) may be necessary for high-risk NMIBC patients to mitigate the risk of recurrence [11], and pembrolizumab has been approved for high-risk patients who are unresponsive to BCG [12]. Approximately 30% of bladder malignancies are MIBC, necessitating more aggressive treatment. Radical cystectomy, traditionally considered the standard for MIBC, is now recognized as being in clinical equipoise with bladder-preserving trimodal therapy (TMT) in appropriately selected patients [13, 14]. In certain patients, TMT may be considered, which entails concurrent radiation therapy, chemotherapy, and maximal TURBT [14]. Adjuvant nivolumab should be considered for patients with advanced MIBC who did not receive neoadjuvant cisplatin-based therapy or for patients who had residual disease at the time of cystectomy. In the event of high-risk pathological features at the time of resection (pT3-4, positive lymph nodes, positive margins), adjuvant radiation therapy may be considered [15].

The preferred front-line treatment for metastatic bladder cancer is the combination of pembrolizumab and enfortumab vedotin [15]. This combination has shown significant efficacy compared to chemotherapy alone, as studies indicate greater rates of progression-free survival and overall survival [16]. Enfortumab vedotin, an antibody-drug conjugate that targets Nectin-4, functions by improving the immune system’s response to tumor cells, whereas pembrolizumab is a PD-1 inhibitor [16]. There are some risks with this treatment, even though it is effective. Most of the patients (55.9%) had side effects that were grade 3 or higher. These included peripheral sensory neuropathy (50%), maculopapular dermatitis (7.7%), hyperglycemia (5%), neutropenia (4.8%), and diarrhea (3.6%) [16]. Because of this, it is important to monitor these patients closely, especially when used with other treatments like radiation.

The CheckMate 274 trial has shown that adjuvant nivolumab has substantially improved the disease-free survival of high-risk MIBC patients following radical surgery, with a median disease-free survival that was nearly doubled compared to the placebo (20.8 vs. 10.8 months) [17]. This discovery underscores the potential of immune checkpoint inhibitors to mitigate the risk of recurrence in a population with restricted post-surgical treatment options. Pembrolizumab has been FDA-approved for high-risk NMIBC patients who are unresponsive to BCG therapy, and immunotherapy has also been extended to NMIBC [17].

The rationale for combining immunotherapy with radiation therapy in bladder cancer is particularly compelling due to the unique tumor microenvironment of urothelial carcinoma, which exhibits a high mutational burden and immune cell infiltration [18]. These characteristics make bladder cancer an ideal candidate for immunomodulation, potentially enhancing the synergistic effects of radiation-induced immunogenic cell death and immune checkpoint blockade [19].

### Current roles of radiation

Radiation therapy is significant in the management of MIBC. Bladder-sparing TMT serves as an alternative for patients who are unsuitable for surgery or who opt for bladder preservation [14]. This approach is recommended for specific instances involving unifocal malignancies, complete resections, and the lack of tumor-associated hydronephrosis or carcinoma *in situ* [20]. In patients who are appropriately selected, TMT has exhibited disease-specific survival rates that are comparable to those of radical cystectomy, providing a viable alternative to bladder excision while preserving quality of life [20]. In palliative care, radiation therapy is implemented to alleviate symptoms such as hematuria or distress that are linked to metastatic cancer [21]. Shorter and more convenient treatment regimens, particularly for elderly patients, have been made possible by advancements in radiation treatments, such as hypofractionated radiation.

The current treatment of bladder cancer is characterized by the distinct functions of radiation therapy and immunotherapy. Consequently, there is a growing interest in the effective combination of these modalities to improve patient outcomes. Radiation therapy has been a fundamental component of localized control in MIBC for an extended period, while immunotherapy has emerged as an instrument for managing advanced and high-risk cases, particularly through the use of immune checkpoint inhibitors. The synergy between these two techniques has great therapeutic value since radiation can change the tumor microenvironment, therefore enhancing the effectiveness of immunotherapy. The aim of this study is to evaluate the present data on combined immunotherapy and radiation. The study will mostly focus on the mechanism, clinical evidence, and challenges in maximizing this strategy.

## MECHANISMS OF ACTION: RADIATION AND IMMUNOTHERAPY

### Radiation’s immunogenic effects

An immunogenic cascade is initiated by radiation therapy, which increases the tumor’s susceptibility to immune responses within the tumor microenvironment. One of the primary mechanisms is radiation-induced cell death, which enhances the visibility of the tumor to the immune system by promoting the release of tumor-associated antigens [22]. The activation of dendritic cells is accompanying this process, which is necessary for the presentation of antigens to T-cells and the subsequent mobilization of the immune system for an anti-tumor response [22]. Moreover, radiation modifies stromal cells and enhances immune cell infiltration, thereby modifying the tumor microenvironment [23]. This entails increasing the recruitment of immune effector cells on endothelial cells by upregulating adhesion molecules including ICAM-1 and VCAM-1 [23]. Radiation can increase immunological activity by triggering the release of pro-inflammatory cytokines, such as interferon-gamma [23]. The combination of immunotherapy and radiation’s immunogenic effects promotes immune-mediated tumor control.

### Immunotherapy mechanisms

Immunotherapy has transformed cancer treatment by augmenting the immune system’s capacity to identify and eliminate cancer cells. The two principal immune checkpoint mechanisms are the PD-1/PD-L1 and CTLA-4 pathways [24]. PD-1 is a receptor found on activated T cells that interacts with its ligands PD-L1 or PD-L2 on tumor cells, resulting in immune evasion [24]. By obstructing this connection, immune checkpoint inhibitors like pembrolizumab and nivolumab enhance T-cell function, enhancing the immune response against tumor cells [25]. CTLA-4 is another checkpoint receptor found on T cells that competes with CD28 for binding to B7 molecules on antigen-presenting cells, hence inhibiting early T-cell activation [25]. Ipilimumab, an anti-CTLA-4 antibody, inhibits this inhibition, thereby increasing the proliferation and activation of T cells [25]. Collectively, these immune checkpoint inhibitors activate the immune system, thereby facilitating the efficient identification and elimination of cancer cells. Consequently, they are effective tools for cancer treatment. Furthermore, often given in combination with pembrolizumab is enfortumab vedotin. By targeting Nectin-4 on tumor cells and delivering cytotoxic chemicals to promote death, enfortumab vedotin increases the immune-enhancing properties of checkpoint inhibitors, hence generating a synergistic effect with pembrolizumab [26].

Although they both work well against bladder cancer, PD-1/PD-L1 and CTLA-4 inhibitors affect the immune system in different ways. Reversing T cell exhaustion and improving the activity of pre-existing tumor-specific T cells within the tumor microenvironment are the main ways that PD-1/PD-L1 inhibitors work [27]. As a result, the immune response is more focused and has a generally positive safety profile [27]. Early in the immunological response, CTLA-4 inhibitors increase naïve and memory T cell activation and proliferation while decreasing Treg-mediated repression [28]. A stronger but less focused immune activation may arise from this, which frequently raises the incidence of immunological-related adverse effects [28].

### Synergistic mechanisms of combining radiation and immunotherapy

The synergistic mechanisms of radiation therapy and immunotherapy have demonstrated significant potential for enhancing the outcomes of cancer treatment. Localized tumor cell death is induced by radiation therapy, which results in the release of antigens that facilitate dendritic cell activation and subsequent T-cell priming. These activated T-cells can subsequently spread to distant tumor sites, causing a systemic immune response known as the abscopal effect [29]. Immunotherapy, particularly immune checkpoint inhibitors such as anti-PD-1 and anti-CTLA-4 antibodies, reactivates exhausted T-cells, allowing them to mount an efficient anti-tumor response [30]. When paired with immunotherapy, radiation therapy can improve antigen presentation as well as immune cell infiltration and activity. This combination not only improves tumor management at the main location, but it also helps to remove distant metastases [30].

Rompré-Brodeur et al. conducted a study indicating that the integration of radiation therapy with anti-PD-L1 therapy in a murine bladder cancer model led to substantial tumor regression at both irradiated and non-irradiated sites, emphasizing enhanced cytotoxic T-cell infiltration and a tumor microenvironment transformation that promotes cytotoxic activity [31]. These findings highlight the potential of integrating radiation and immunotherapy to augment systemic antitumor responses and increase outcomes in patients with metastatic bladder cancer.

One of the most difficult hurdles in cancer treatment is overcoming immunotherapy resistance, which is often caused by immune evasion mechanisms in the tumor microenvironment. Beyond immune cells, the microenvironment includes cancer-associated fibroblasts, extracellular matrix elements, and endothelial cells, all of which contribute to immune evasion and tumor growth [32]. Cancer cells consume an increased amount of glucose as a result of metabolic changes, including the Warburg effect, which restricts the availability of glucose and impairs T cell function [32]. Additionally, the microenvironment is acidified by lactic acid accumulation, which enhances hypoxia and stabilizes hypoxia-inducible factor. This process stimulates angiogenesis while simultaneously decreasing immune cell infiltration and activity [32].

This microenvironment can be modulated by radiation therapy, which can assist in the surmounting of resistance. It accomplishes this by augmenting the recognition of tumor cells by cytotoxic T-cells and increasing the expression of major histocompatibility complex class I (MHC-I) molecules [30]. Furthermore, radiation induces the secretion of pro-inflammatory cytokines and activates pathways such as cGAS-STING, which in turn promotes the activation of dendritic cells and the production of type I interferon [30]. These modifications establish a more favorable immune environment, which facilitates efficient functioning of checkpoint inhibitors. Furthermore, radiation has the ability to reverse tumor immune evasion by downregulating inhibitory molecules such PD-L1, which are typically overexpressed in resistant cancers [33]. Radiation has the ability to convert previously resistant malignancies to immune-responsive ones by altering immune-suppressive components, making tumors more susceptible to immunotherapy [33].

Although these insights offer a compelling biological justification for the integration of radiation treatment and immunotherapy, clinical evidence is essential for substantiating these effects in practice. Numerous clinical trials have investigated the safety and efficacy of this combination in bladder cancer, providing significant data on patient outcomes and treatment viability.

## CLINICAL DATA

As we look at the clinical data (Table 2), this section aims to give a summary of important trials that test the safety and effectiveness of different combinations of immunotherapy and radiation therapy in bladder cancer. The studies presented range from early-phase investigations to larger trials, each of which investigates the use of various immunotherapeutic agents, including PD-1 and PD-L1 inhibitors, in conjunction with radiation. These agents are used either as monotherapy or as part of a trimodal approach which includes chemoradiation.

**Table 2:** Summary of trials investigating the combination of immunotherapy and radiation therapy in bladder cancer

Trial	Phase	Patient population	Intervention	Primary endpoints	Key findings	Toxicity
**BTCRC-GU15-023**	II	T2-4 N0-2 M0 bladder cancer, cisplatin-ineligible	Durvalumab with radiation therapy	Disease control, PFS, OS	72.7% achieved disease control; 54.5% complete response. Median PFS: 21.8 months; OS: 30.8 months.	Well-tolerated; some cases of anemia, high lipase/amylase levels, immune-related nephritis, no radiation-specific immune toxicities
**Cuellar et al.**	II	Localized MIBC (T2-4a N0 M0), bladder preservation option	Durvalumab plus Tremelimumab with radiotherapy (46 Gy pelvis, 64-66 Gy bladder)	Pathological response (≤T1)	Trial expanded to second cohort after meeting activity goal; demonstrating feasibility of bladder preservation	No severe immune-related toxicities reported
**INTACT**	III	Localized MIBC (T2-T4a N0 M0)	Atezolizumab with concurrent CRT (TMT plus atezolizumab vs. TMT alone)	Treatment response, recurrence, survival	Preliminary data shows comparable safety, no significant increase in immune-related toxicities, DSMC recommends continuation	Higher rate of hematological grade 3 AEs in atezolizumab group, minor radiation cystitis
**NEXT**	II	Localized MIBC, post-TMT	Nivolumab as adjuvant after TMT	2-year FFS, local control, cystectomy rates, OS	Investigating enhancement of immune response post-TMT for improved survival and control	Ongoing; safety data pending
**ANZUP**	II	MIBC, bladder preservation or cystectomy-ineligible	Pembrolizumab with chemoradiotherapy (64Gy over 32 fractions, cisplatin)	Safety, complete response rate	Focus on evaluating bladder-sparing potential and safety for localized MIBC	Grade 3–4 adverse events; ongoing
**Marcq et al.**	I/II	MIBC, predominantly cT2	Atezolizumab with TMT (TURBT, IMRT, gemcitabine)	Toxicity	Study halted due to unacceptable toxicity; grade 3 toxicities in 37.5% of patients	No grade 4 AEs or deaths; high rate of grade 3 toxicities, leading to premature termination of study
**PLUMMB**	I	Metastatic or locally advanced bladder cancer	Pembrolizumab with weekly hypofractionated radiation therapy (36 Gy in 6 fractions)	Tolerability	Dose-limiting toxicities in initial cohort, including severe grade 3 urinary toxicities and a grade 4 rectal perforation	Significant grade 3 urinary toxicities, grade 4 rectal perforation, trial paused and recommended dose reduction

Abbreviations: AEs: Adverse Events; CRT: Chemoradiotherapy; DSMC: Data and Safety Monitoring Committee; FFS: Failure-Free Survival; IMRT: Intensity-Modulated Radiation Therapy; OS: Overall Survival; PFS: Progression-Free Survival; TMT: Trimodal Therapy.

### BTCRC-GU15-023

Joshi et al. performed a phase II study to determine the safety and efficacy of combining radiation therapy with durvalumab, a PD-L1 inhibitor, in patients who were ineligible for surgery or cisplatin-based chemotherapy [34]. This multi-institutional, single-arm experiment included 26 patients with T2-4 N0-2 M0 bladder cancer and yielded encouraging results. The majority of patients (72.7%, 95% CI 49.8–89.3) achieved disease control post-adjuvant therapy, while 54.5% had a complete response, with a median follow-up of 27 months. The progression-free survival (PFS) rate was 71.5% (95% CI 55.6–91.9%) at one year, with a median PFS of 21.8 months (95% CI 14.8–not reached) and an overall survival (OS) rate of 30.8 months (95% CI 22.9–not reached). Significantly, the combination was well-tolerated, with the most prevalent treatment-related adverse events being fatigue (57.7%), decreased lymphocyte count (46.2%), diarrhea (38.5%), cystitis (34.6%), maculopapular rash (23.1%), and urinary tract infection (23.1%). However, no additional immune-related toxicities were observed as a result of radiation. This trial emphasizes the potential advantages of immunotherapy in conjunction with radiation for patients who are ineligible for cisplatin.

### Marcq et al.

The study included eight patients, predominantly with cT2 MIBC, who were treated with TURBT, intensity-modulated radiation therapy (50 Gy over 20 fractions), gemcitabine, and atezolizumab [35]. The trial’s goal was to assess the toxicity of atezolizumab and chemoradiation administered simultaneously. Findings revealed that 60% of patients administered the elevated dosage of atezolizumab encountered grade 3 adverse events, necessitating a dose decrease. Notwithstanding the decrease, grade 3 toxicities continued to manifest, comprising colitis (25%), proctitis (12.5%), lymphopenia (12.5%), neutropenia (12.5%), acute renal damage (12.5%), and elevated GGT (12.5%). As a result, the investigation was prematurely concluded. There were no fatalities or adverse events classified as grade 4. The study concluded that the concurrent administration of atezolizumab and TMT in this context resulted in an intolerable toxicity profile, suggesting the need for alternative treatment combinations in bladder-preserving regimens for MIBC. This investigation emphasizes the challenges associated with multimodal combination therapies, including immunotherapy and chemoradiation, in the treatment of bladder cancer.

### PLUMMB

A phase I study was conducted to assess the tolerability of combining weekly hypofractionated radiation therapy (36 Gy in 6 fractions) with pembrolizumab in patients with metastatic or locally advanced bladder cancer [36]. Five patients participated in the experiment, and pembrolizumab was provided two weeks before radiation therapy. Unfortunately, the initial dose cohort exhibited dose-limiting toxicity, with two of the five patients suffering from severe grade 3 urinary toxicities attributable to the treatment, and one patient experiencing a grade 4 rectal perforation. The radiation therapy dosage was advised to be decreased, and the trial was suspended. This trial highlights the risks associated with the combination of immune checkpoint inhibitors and high-dose hypofractionated radiation in pelvic malignancies, emphasizing the importance of careful dose management. Future study may necessitate the exploration of decreased radiation doses or premedications to mitigate off-target immune system activity when investigating these combinations.

### Cuellar et al.

This phase II study by the Spanish Oncology Genitourinary Group (SOGUG) investigates the efficacy and tolerability of the combination of the anti-PD-L1 monoclonal antibody durvalumab, the anti-CTLA-4 antibody tremelimumab, and radiotherapy for bladder-sparing therapy in patients with localized MIBC [37]. The objective of the trial is to assess the feasibility of bladder preservation in patients with clinical stages T2-4a N0 M0 who either elect for bladder preservation or are ineligible for cystectomy. The primary endpoint is pathological response, which is defined as a T1 or lower on the post-treatment biopsy. The study adopts a two-stage sequential design, with the main target of achieving at least six responses from the first 12 patients before moving on to a second cohort of 20 patients. The study was successfully progressed to the second stage after completing this activity aim in December 2019. The treatment protocol consists of transurethral resection, immunotherapy (durvalumab and tremelimumab every 4 weeks for three dosages), and normofractionated external beam radiotherapy (46 Gy to the pelvis and 64-66 Gy to the bladder). Although specific toxicity data has not yet been provided, the trial’s ongoing evaluation encompasses the assessment of both radiation-associated and immune-related adverse events. This investigation is particularly pertinent for illustrating the potential of combining immunotherapy with radiotherapy to preserve the bladder in MIBC.

### INTACT

The INTACT trial is a crucial phase III randomized study that assesses the safety and efficacy of concurrent chemoradiotherapy (CRT) with or without the anti-PD-L1 immune checkpoint inhibitor atezolizumab in patients with localized muscle invasive bladder cancer [38]. The aim of this experiment is to determine if the incorporation of atezolizumab into TMT improves patient outcomes. The primary endpoint was the treatment response, which was evaluated through biopsy three months after CRT, and the secondary endpoints included recurrence and survival. A total of 475 patients with MIBC (T2-T4a N0 M0) were randomized. At the time of this interim analysis, the primary endpoint results were not yet accessible. In the safety analysis of the initial 73 patients, 37 patients received TMT in conjunction with atezolizumab, while 36 patients received TMT alone. 23 (62%) grade 3 or higher toxicities were observed in the atezolizumab group, while 11 (31%) were observed in the non-atezolizumab group, according to the study. These were primarily hematological and not connected to the immunological system. Following atezolizumab treatment, only one patient experienced grade 3 radiation cystitis. The Data and Safety Monitoring Committee (DSMC) advised the continuation of enrollment based on these findings, indicating that the incorporation of atezolizumab did not significantly elevate immune-related toxicity. The safety results from this study is crucial for the continued integration of immunotherapy with CRT in MIBC.

### NEXT

The NEXT trial is a phase II, open-label investigation that is designed to evaluate the efficacy of nivolumab, a PD-1 checkpoint inhibitor, as an adjuvant therapy after TMT in patients with localized MIBC [39, 40]. In MIBC, TMT carries a local recurrence risk of 11 to 18% within the first five years, emphasizing the potential for disease resurgence following treatment [41]. The concept underlying this experiment proposes that nivolumab will augment the tumor-specific immune response elicited by chemoradiation, potentially enhancing failure-free survival (FFS) both locally and systemically. Participants in this research were administered nivolumab within 90 days of finishing TMT. The principal goals of the study comprise 2-year FFS, local control, radical cystectomy rates, distant FFS, overall survival, and quality of life. As of the data cut-off, the 2-year FFS was documented at 38.7% (95% CI 23–65.2%). Sixteen individuals experienced disease relapse, with nine suffering from local recurrences. The interim analysis did not provide specific percentages for various adverse occurrences. Nonetheless, grade ≥3 treatment-related adverse events manifested at an overall incidence of 10.7%. This single-arm trial is notable as it aims to extend the advantages of immunotherapy to patients who have completed chemoradiation, perhaps improving both local control and abscopal effects in bladder cancer.

### ANZUP

The purpose of the ANZUP trial, a pilot phase II study, is to assess the safety and viability of using pembrolizumab in conjunction with chemoradiotherapy for MIBC patients who either prefer bladder preservation or are not eligible for cystectomy [42]. Thirty patients, recruited from several sites around Australia, are undergoing a regimen of 64Gy of radiation therapy over 32 fractions, concomitant with cisplatin and pembrolizumab. Safety is the primary endpoint of the trial, as defined by the incidence of grade 3–4 adverse events or failure to complete therapy. Efficacy is the secondary endpoint, defined as the rate of complete response at 12 and 24 weeks post-treatment, as determined by cystoscopy assessments. The trial reported a complete response rate of 88% at 24 weeks, a 2-year locoregional progression-free survival rate of 87% (95% CI 64–96%), and a distant metastasis-free survival rate of 78% (95% CI 54–90%) with a median follow-up of 31 months. The median overall survival was 39 months (95% CI 17.1–not evaluable). Nine participants (32%) experienced predefined grade 3 or worse adverse events, including one treatment-related death due to respiratory failure. The results of this trial provide evidence that the combination of pembrolizumab and chemoradiotherapy is feasible, showing promising response rates.

## CHALLENGES AND FUTURE DIRECTIONS

The absence of large-scale randomized controlled trials is one of the primary obstacles to the integration of immunotherapy with radiation therapy in bladder cancer. This limitation restricts the ability to draw definitive conclusions about the efficacy and safety of this combination, particularly in the context of the management of overlapping toxicities. Radiation can exacerbate immune-related adverse events such as pneumonitis, colitis, and dermatitis, complicating treatment regimens [43]. It may also trigger a pro-inflammatory tumor microenvironment, which can both promote immune activation and heighten the risk of immune-mediated toxicities, such as lymphopenia [43]. The immune-suppressive mechanisms, designed to improve tumor recognition by the immune system, may also lead to detrimental side effects when radiation is administered alongside immunotherapy. These variables highlight the necessity for randomized controlled trials to confirm the synergistic potential of radiation and immunotherapy, while also addressing toxicity management.

To reduce overlapping toxicities, careful monitoring is needed when integrating immunotherapy with radiation therapy. Fractionated radiation treatments and other optimized dose schedules can limit toxicities and excessive immune activation. For example, because of documented dose-limiting urinary toxicities, the PLUMMB study emphasized the necessity to lower the radiation therapy dose per fraction when coupled with pembrolizumab [36]. This suggests that fractionated regimens may be more safe. Another approach is to change the immunotherapy sequencing. To decrease inflammatory reactions and associated toxicities, neoadjuvant or adjuvant immunotherapy is generally preferred to concurrent dosing. According to Daro-Faye et al., neoadjuvant or adjuvant immunotherapy is suggested in localized MIBC due to safety concerns associated with simultaneous hypofractionated radiation therapy [43]. Furthermore, cytokine profiling or T-cell exhaustion signs can be employed in biomarker-driven patient classification to predict and avoid immune-related adverse events [44, 45].

Despite mounting evidence of the clinical equipoise between surgery and TMT [13, 14], a significant drawback of current adjuvant immunotherapy trials is their emphasis on patients undergoing radical cystectomy. This has created an absence of data on whether immunotherapy offers comparable advantages to individuals receiving TMT and further research is needed to assess adjuvant immunotherapy in this setting.

A significant question pertains to the identification of patients who would derive the greatest benefit from immunotherapy following radiation treatment. Bladder cancer exhibits diverse genetic profiles, and patient responses to immunotherapy may vary, particularly after radiation-induced alterations in the tumor microenvironment, which can either amplify or worsen immune-related toxicities. Radiation therapy can activate immunological pathways, including the cGAS-STING pathway, enhancing T-cell priming; however, it may also recruit myeloid-derived suppressor cells and regulatory T cells, creating an immunosuppressive environment [46]. Identifying biomarkers such as PD-L1 expression, tumor mutational burden, and immune infiltration levels may enhance patient classification and the customization of therapy options [46]. However, there is no agreement on the best criteria for patient selection. Tailoring treatment for those most likely to benefit from immunotherapy after radiation, while limiting the possibility of significant overlapping toxicities, remains a key unresolved issue. This necessitates more investigation of prognostic markers, greater characterization of the tumor immune microenvironment, and the development of ways to reduce toxicity during combination therapy.

## CONCLUSIONS

This review emphasizes the significant therapeutic benefits of combining radiation therapy with immunotherapy in bladder cancer treatment. Despite the promising early-phase results, large-scale randomized controlled trials are required to evaluate the safety and efficacy of this combination, particularly in terms of reducing overlapping toxicities. The lack of agreement on biomarkers, such as PD-L1 expression and tumor mutational burden, highlights the need for more investigation to enhance patient classification and tailor treatment. Further study should focus on identifying predictive biomarkers, improving toxicity management, and exploring tailored treatment approaches in order to maximize the benefits of this combination in clinical practice.
